# Skills and key education needed for clinical librarians: an exploratory study from the librarians' perspectives

**DOI:** 10.1186/s12911-021-01601-y

**Published:** 2021-08-09

**Authors:** Maryam Zarghani, Leila Nemati-Anaraki, Zahra Dinpajoo, Arezoo Ghamgosar, Sedegheh Khani, Maryam Khazaee-Pool

**Affiliations:** 1 School of Health Management and Medical Information Science, Iran University of Medical Sciences, Tehran, Iran; 2 Department of Medical Library and Information Sciences, School of Health Management and Medical Information Science, Iran University of Medical Sciences, Tehran, Iran; 3 Department of Public Health, School of Health, Mazandaran University of Medical Sciences, Sari, Iran; 4 Health Sciences Research Center, Addiction Research Institutes, Mazandaran University of Medical Sciences, Sari, Iran

**Keywords:** Clinical librarianship, Clinical Librarian—specialized skills, Professional skills

## Abstract

**Background:**

A clinical librarian is a member of the medical team in many countries. To strengthen this new job, librarians need to acquire professional skills in order to provide information services to medical staff. In this study, we aimed to explor the skills required for the presence of a clinical librarian in the treatment team.

**Methods:**

In this study, we sonducted a qualitative study in which 15 experienced librarians were interviewed in connection with information services. Also, a treatment team was involved in this study using purposive-convenience and snowball sampling methods. The data collection tool was a semi-structured interview that continued until the data was saturated; finally the data analysis was performed using thematic analysis.

**Results:**

Out of the total interviews, 158 primary codes and, 107 main codes were extracted in 25 subclasses. After careful evaluation and integration of subclasses and classes, they were finally classified into 13 categories and four main themes, namely clinical librarian’s role, professional and specialized skills, communication skills, and training programs.

**Conclusion:**

The results showed that specialized skills and training programs for the clinical librarian are defined based on his/her duties in the treatment team. We also defined the most important key skills for the clinical librarian in two categories of professional and communication skills such as specialized information search, content production, resource management, familiarity with various sources related to evidence-based medicine, teamwork, and effective communication. To acquire these skills, officials and policy-makers should develop and implement related educational programs at medical universities and colleges.

**Supplementary Information:**

The online version contains supplementary material available at 10.1186/s12911-021-01601-y.

## Background

The unlimited increase in information resources has left health professionals with a vast amount of information. Thus, it is difficult for them to identify and select the best information [1], and to implement evidence-based medicine that focuses on issues such as lack of time to search and information retrieval [2, 3]. To improve the quality of patient care, the treatment team needs to identify treatment and diagnostic methods based on the best evidence [4]. Librarians' support for the care team has led to major benefits such as saving physician’s time, reducing costs, supporting decision-making, and improving health care [5]. Accordingly, medical staff needs specialized and trained clinicians to retrieve clinical information [6].

A *Clinical Librarian* is a professional and capable person who is effective in providing the information needed of medical and research team members [7], and has a key role in supporting clinical decisions [2]. During 1971–1973, Lamb launched the first clinical librarianship program at University of Missouri, Kansas, sponsored by the National Library of Medicine Lamb believed that librarians who had been trained in information retrieval skills should be active members of the treatment team [8].

The importance of the clinical librarian in evidence-based treatment and medicine processes has been reflected in many studies. Cimpl explained that clinical librarian programs provide information to medical staff quickly, influence the information retrieval behavior of physicians, and improve their library skills [6]. Lawton and Burnes found that constant updating of specialized skills in order to achieve emerging health roles is a necessary skill for being a health librarian [9]. Also, Zebliski et al. [10] considered the following as vital for health librarians: increasing self-confidence and specialized knowledge, ease of access to resources, improving skills in using new information technologies, and the ability to use databases in order to improve the librarians’ ability to participate in evidence-based medical programs [10].

In addition, Sen et al. found that the most important roles for librarians in the field of health were education, information retrieval, and management [11]. To accept these roles, they must acquire specialized information technology and skills management. Meanwhile, Harrison explored new librarians' roles in health and their key education and skills [12]. Ozan [13] described librarians in the field of health as consumers of information. To Ozan, the function of information consumption is the promotion and teaching of health literacy, educating patients and organizations, and distribution of educational materials. Also, Regan et al. [14] investigated the supportive role of clinical librarianship in optimizing real-time evidence-based radiotherapy processes. Meanwhile, there is a close relationship between the role of a clinical librarian and evidence-based medicine, which is a systematic process that examines, evaluates [15], and uses the best evidence in clinical decisions made correctly and wisely in order to better care of patients [6]. The expansion of the evidence-based medicine and providing accurate and timely information to the members of the healthcare team necessitate the presence of a clinical librarian in the team [16].

For the successful presence of librarians in the treatment team, they should learn the required skills and receive professional trainings regularly based on the academic educational programs so that the quality of medical services will improve. One of the best ways to identify these needs is to understand and analyze librarians' experiences who have worked in a clinical team or conducted research in clinical librarianship. To this end and given the important role of clinical librarians in the medical teams, we in this study explore the perspectives and experiences of librarians who are active in medical settings. In doing so, we analyze the educational programs and specialized communication skills required for clinical librarians in the medical teams. We hope that results of this study could help officials involved in education and librarianship to develop the necessary educational programs.

## Methods

### Participants

This is a qualitative research study. The population in this study includes librarians working in hospitals and clinical research centers in Tehran. All the participants graduated in librarianship and medical information with more than 5 years of work experience or cooperation with treatment teams with at least one research study. The sampling method in the first stage was purposive convenience sampling. After selecting the primary sample units, we used the snowball method in order to identify other eligible individuals. The research environment was hospital libraries, clinical research centers, and the participants’ workplace selected based on the participants’ suggestions.

### Data collection

We used semi-structured interviews to collect data. To conduct the interviews, we used the same studies that were used in preparing the initial research design. Also, we used the opinions of the research team. Then, we prepared the interview guide. During the interview with three participants in the pilot phase, the number of items, the time of the interview, and the clarity of the items were decided. We then finalized the questions. More details about interview questions are given in Additional file 1. The interview started with a general question about the participant’s working conditions and their experiences of working in a clinical setting. We narrowed down the interview to more specific questions based on research objectives. After determining the time and place/medium of the interview, we sent the interview guide to the participants via email. Interviews were conducted using three methods: face-to-face, telephone, and online. The approximate time for each interview was 30 to 45 min. We continued the interviews until we felt that the data was saturated. Overall, we conducted 15 interviews. When we reached the data saturation, we were ensured that there would not be more new data related to the research topic by conducting more interviews.

### Data analysis

We performed the data analysis based on thematic analysis. At the end of each interview, we meticulously transcribed the interview data. Also, one of the researchers listened to the recorded interviews. In the next step, the primary codes were determined. Meanwhile, we conducted text transcription manually using Microsoft Word software. To control the validity of the data, we used the method of agreement between the three coders (three members of the research team) who coded the first three interviews in parallel, and then discussed to agree on the codes. In addition, the method of reviewing by participants in the interview was used to determine the validity and reliability of the data. In the next step, we sent a part of the transcribed text with the primary codes to a group of participants in order to compare and confirm the consistency of the ideas that emerged from the data. At the end of the interviews, we re-examined the transcription of the interviews, and then merged the similar codes and sub-classes. Next, we found the connection between the subclasses and categorized them into larger classes; this way, we were able to extract the main themes of the study. The entire coding and integration processes were performed under the supervision of the entire research team.

## Results

Out of the 15 participants in this study, five had a research study with work experience and 10 had experience working with the medical team and the hospital library (Table 1). The initial classification of codes started from the first interview to form classes and subclasses. In subsequent interviews, the codes of each interview were compared with each other and with the other codes of previous interviews in order to determine their similarities and differences. Then, the codes were placed in a certain class based on their similarities with each other. Also, we reviewed and compared classes, which had a significant impact on the development of classes and the placing of codes in an appropriate class. This further caused some classes to merge or create newer classes, as data collection and analysis continued. Finally, 158 primary codes, 107 main codes and 25 sub-classes, 13 classes, and four main themes were extracted (Table 2). The main themes include the clinical librarian's role, specialized skills, communication skills, and educational programs required for the clinical librarian. Based on these themes, we extracted the description of the sub-classes according to the following process.

**Table 1:** The participants’ information

Number	Job title	Work experience
P1	Hospital librarian	10 years of experience
P2	Hospital librarian	2 years as a clinical information assistant and one research project
P3	Hospital librarian /professor/ researcher	13 years of work experience and two research projects
P4	Hospital librarian/professor/researcher	17 years of work experience and four research projects
P5	University librarian and hospital librarian/researcher	3 years of teaching experience and two research projects
P6	Librarian of the clinical research Center	9 years of experience
P7	Hospital librarian—professor–researcher	7 years of experience and 14 years teaching
P8	Professor–researcher	30 years of teaching and work experiences and two research projects
P9	Hospital librarian	5 years of experience
P10	Hospital librarian	9 years of experience
P11	Librarian of the clinical research center	5 years of experience and two research papers
P12	Hospital librarian	10 years of experience
P13	Hospital librarian/student of LMI/researcher	5 years of experience and one research work
P14	Librarian of the clinical research center	8 years of experience
P15	Hospital librarian/teacher/researcher	20 years of experience and two research

**Table 2:** Themes and subcategories extracted for skills and trainings of clinical librarians

Theme	Category	Semantic unit
The role of the clinical librarian	Information consultant of the clinical team	“I believe that medical librarians, along with physicians, can gather information and give information-related advice, because usually, we are good collectors, we find things that a normal person cannot find, and besides, we are also good information critics who can monitor which information is useful and which is not, which is valid and which is not..”
	Information search specialist	“A librarian is successful who as a member of the team (along with assistants, physicians, and medical professionals), can teach the clinical team and meet their information needs by searching for articles, and teaching how to search
	Health information and information literacy in the treatment department	“Librarians, along with the doctor, can prepare educational content by searching for appropriate and pure information. In this content and educational package, the patient should know about his disease, what treatment he has, and what nutrition he should have. And what are the side effects, etc.”
Specialized and professional skills	Critical appraisal of various sources of information	“Well, the ability to critically appraise medical information from articles, evaluate medical texts, the ability to use evidence-based medicine along with specialized search skills and technology is necessary for a clinical librarian”
	Familiarity with medical databases	“Complete familiarity with databases, reputable medical sites, especially sites that are related to associations and provide items such as medical guidelines, and how to search and access information that is an important part of information providing process”
	Familiarity with search strategies	“Know how to work with databases so that you can achieve the best results in the shortest possible time. Databases such as Scopus, PubMed, mastering these training is of top priority, but before that mastery of terminology is critical, and if I know the basics well but do not know the terminology, I can not be very effective”
	Familiarity with the new information technologies in the field of medicine	“I think a clinical librarian should be familiar with ICDL skills in such a way that if an online conference is offered to doctors if there is a problem in the network and internet in the middle of the conference, the librarian could quickly troubleshoot. Also, the librarian should be proficient in modern technologies in the profession and increase his knowledge in this field”
	In-depth knowledge of clinical terminology and applied English related to the clinical environment	“In the first step, sufficient knowledge of English and possibly fluency in English, in my opinion, is an important step in this field, but in the meantime, sufficient knowledge and mastery of medical terms should not be neglected. When starting in a hospital environment most clients who were clinical staff used English words frequently in their conversations to express what they needed, and since I took English classes before college, this skill was very useful and helped me*"*
Communication and interaction skills	Acquiring appropriate interaction and communication skills	"We have to be patient. Be nice to others. It was interesting one of the doctors said he would go to a librarian with a smile on her face more willingly than a librarian with a frown sitting on her back with no contact. It helps me to better trust and addresses the doctor’s need for information. In fact, the type of communication the librarian has encourages doctors to keep asking.
	Having motivation and confidence to work in a clinical setting	"I think personal characteristics are also important. If you have the motivation to work in the environment, that motivation will help you to survive the hardships of the clinical environment, and this is an intrinsic motivation in each person that makes a difference"
Educational programs	Incorporating new courses into the curricula	“I think the biggest problem our librarians have is the courses that are taught at the university, they need to be revised. These courses do not teach students how to become clinical librarians, and should be replaced with courses that meet professional requirements” “The specialized Librarianship courses of the university should be fundamentally revised and the chapters should be revised according to the need for work"
	Passing an internship in the clinical team	"This should be practically so that it is done in the form of an internship in the hospital environment, which eliminates the students' fears and increases their self-confidence, and it is necessary for the professors who are fluent to do this along with students."
	Training of professional-communication skills for working in a clinical environment	"I think you need computer skills, intermediate to advanced English language skills, mastery of medical terminology, verbal skills, and psychology lessons to better understand people and how to approach and absorb them. Because of the diverse nature of referrals and search engines, search skills and access to information are required for training programs."

### Theme 1: The role of the clinical librarian

This theme counted four sub-themes including information consultant of the treatment team, information retrieval specialist, health information trainer, and information literacy in the treatment department. Determining the role of the librarian in the treatment team is an important part that can solve many ambiguities regarding the skills and characteristics required for a clinical librarian. Also, by determining the different aspects of this position, the necessary educational programs in the form of university education and continuing education for librarians will be clearer. The first characteristics considered by the participants with the role of a clinical librarian was the information consultant of the treatment team, which includes more specific roles such as consulting, providing the most appropriate information, and a physician’s information consultant.

One of the participants stated:

“Librarians, along with physicians, can provide relevant educational information by seeking appropriate information that includes what the patient needs to know about the disease” (10 years experience, P1).

Understanding and accepting these roles lead to the emergence of another task for the librarian, a prescriber of health information for patients which requires attending morning rounds and performing a specialized search for the clinical team in the process of treating patients:

"Prescribing health information means providing the appropriate amount of information with the patient according to the patient's condition." (10 years experience, P1).

Another participant believes that:

"The clinical librarian must participate in the morning rounds and sometimes cooperate with the physician in making the final decision at the time of diagnosis or in changing the treatment method" (3 years of teaching experience and two research projects, P5).

Due to the development and increase of information resources and technologies, the clinical librarian should also have another task as a health information educator to patients and information literacy to the treatment team. Playing this role in the clinical team will improve patients’ health literacy and also medical staff’s information literacy. To perform this role, the librarian must be familiar with the specialized search methods for reliable information and information sources, new information technologies, and the production of information content.

One librarian commented on this role:

"Librarians, along with the doctor, can prepare educational content by searching for appropriate and pure information. In this content and educational package, the patient should know about his disease, which treatment he receives, and what nutrition he/she should have, and what the side effects are, etc. (Ten years of experience. P1).

The role of the clinical librarian is derived from the duties of the librarian in the treatment environment and the clinical team. The roles that have been approved in this study are as follows: clinical team information consultant, information retrieval specialist, prescribing health information to patients, attending mornings, health information trainer, information literacy in the clinic, and producer of training packages which should be under the supervision of a physician.

### Theme 2: Specialized and professional skills:

Theme number 2 summarized three sub-themes including critical evaluation of various information resources, complete mastery of new information resources and technologies in the field of medicine, gaining in-depth knowledge of clinical terms, and English language proficiency.

Specialized and professional skills in any job position are of special value for gaining credit. In the same vein, clinical librarianship must be proficient in the specialized skills related to the field to gain the trust of the treatment team and patients. These skills require constant training and updating in order to cooperate with the clinical team, and can help librarians and educational administrators in this field to improve educational programs and job skills. To participants, critical evaluation of various sources of information is the first and most important skill, which is the product of critical evaluation of knowledge and creative problem-solving thinking.

One of the participants Saied that:

"Familiarity with evidence-based medical databases, health information literacy, critical thinking in problem-solving, communication principles, information resource management, references, types of research methodologies, basic medical concepts, application of new information technologies in medicine, and internships are things that can be taught both theoretically and practically" (13 years of experience with two research projects, P3).

Another participant stated:

"Clinical librarians are completely familiar with databases, reputable medical websites, especially those related to organizations. Also, they provide items such as medical guidelines, searching, and accessing information that are an important part of the process of providing information." (Eight years of experience, P14).

Full mastery of search methods in various information sources and new information technologies in the field of medicine will strengthen the librarians’ role in the treatment team and provide them with more effective services. To complete these skills, the clinical librarian must have a deep knowledge of clinical terms and English language which would ultimately improve continuing medical education in the field of information resources and retrieval skills in the clinical team.

Another participants said:

"The skills related to information search are effective communication with the treatment staff, fluency in English, the ability to critically appraise medical information from articles, evaluating medical texts, using evidence-based medicine, and storage and retrieval tools." (20 years of experience with two research projects, P15).

### Theme 3: Communication skills:

This theme is summarized into three sub-themes including proper interactive communication, motivation, and self-confidence in order to be able to work in a clinical environment.

The treatment environment is stressful. Team spirit and proper interactions with different people is one of the factors of occupational success in medical settings. In addition to specialized skills, the clinical librarian must have appropriate and strong communication skills to be able to survive with the team and the treatment environment. These skills include effective communication with the treatment staff, motivation to work in the treatment environment, and constructive interaction with the treatment staff. From the participants' point of view, learning appropriate interaction and communication skills is the result of interacting with different people, accepting the conditions of the treatment team, and tolerating different reactions of team members.

According to one of the participants' view:

"It is very important to be polite and to have a common language. It solves many problems and medical terminology is the common language." (10 years of experience, P1).

Another participant also stated:

"Communication skills are very important in how you openly gain the trust of the other party. Communication skills and professional ethics should be added to the courses." (Nine years of experience, P6).

Meanwhile, personality traits of individuals are very important in being successful at work. A clinical librarian must have high motivation and self-confidence in order to be able to work effectively in a clinical environment. Thus, characteristics such as being eager to perform tasks, having a team spirit, suitable interaction with physicians, and patience are very important.

"We have to be patient, and be nice to others. It was interesting for me when one of the doctors said he would go to a librarian with a smile on her face more willingly than a librarian with a frown sitting on her back with no contact.” (Nine years of experience, P10).

From the participants' point of view, having appropriate communication skills and creating a common language for providing information services and teamwork skills are important abilities that a clinical librarian must acquire in order to succeed and gain the trust of the clinical team.

### Theme 4: Training programs:

This theme is summarized to three sub-themes as follows: Inclusion of new courses in training programs, training in the clinical team, and training of professional-communication skills in order to be able to work effectively in a clinical environment.

University and in-service training programs are a very important part for training and promoting clinical librarian skills. Providing trainings based on real and practical professional needs has not been included in the training programs so far; thus, clinical librarians are mainly trained through the official training programs at medical universities. In this regard, programs should provide regular curricula with appropriate contents and courses tailored to job needs of clinical librarians; this way, they can receive appropriate feedback from training programs.

One of the librarians stated:

"I think that the biggest problem our librarians have is the courses that are taught at the university. These courses do not teach students to become a very good clinical librarian. At most, such education would only teach them some medical and clinical terms during their Bachelor’s and Master’s courses.” (17 years of experience and four research projects, P4).

The most important point for the participants is to include new courses in the educational programs and to review the university curriculum. Courses such as treatment information and prescribing health information, teaching clinical content production, and applied English appropriate to the clinical environment should be added to the current curriculum. Meanwhile, in-service training programs should be changed.

One participant believed that:

"We should have a health information prescribing course, in which we teach how to prescribe health information from scratch, how to find the right information packages, and in what format that would meet the patients’ needs.” (10 years of experience, P1).

Internship in a clinical team under the supervision of an expert instructor is another important part of the training that prepares librarians to cooperate with the treatment team. Passing this course improves the skills and application of the knowledge gained from the training programs. Integrating the skills acquired from university courses and applying them into practice in response to the needs of the treatment team will familiarize the members of the treatment team with the roles and support of the librarians. Also, it will enhance the communication skills of librarians which is required for taking an active part in the treatment team.

One of the participants who had experience working with the treatment team said:

"We need teaching the skills which are at the heart of our curriculum that do not currently exist in our courses, such as evidence-based medicine and communication skills for librarians. That should be taught at university. Alongside, librarians in the workplace should be persistent in the retraining and updating them." (13 years’ experience and two research projects, P3).

Another librarian reported that:

"In my opinion, in the university environment, they should first be familiar with the various types of databases for evidence-based medicine, and then medical terminology. I think they should increase the number of medical terminology courses." (2 years of experience and one research project, P2).

## Discussion

The presence of an information specialist in the treatment teams has been supported by numerous theories and studies; and this has also been confirmed to have a positive correlation with improving the treatment processes. The clinical librarian has been proposed as an active member of the team that supports evidence-based medicine in clinical settings by providing prompt specialized and quality information with physicians and other members of the health care team [4, 12, 14], influencing physicians' information retrieval behavior and improving their library skills [13, 17], teaching and facilitating access to scientific texts and resources [18], attending morning rounds and reports, being constantly available for information counseling, supporting continued medical education, and patient education [19, 20].

Although the presence in clinical teams has led to the improvement of treatment processes, it does not mean that every information specialist in the clinical team will improve this process. Rather, it requires the acquisition of professional skills in the form of training courses based on the tasks defined in the clinical team. This study examined the professional skills and trainings required for clinical librarians through exploratory analysis of the content of interviews with librarians who had work experiences in medical settings or conducted research in the field of clinical librarianship. The identified themes and subcategories based on the clinical librarians’ tasks in the treatment team identified the skills and trainings needed to create and develop these roles. The duties of a clinical librarian in a treatment team include seeking specialized information and teaching health information and literacy to patients. These duties depend on the university educational programs and continuing education courses.

In the related literature on this topic, the required skills of clinical librarians are as follows: familiarity with statistical principles, critical evaluation of evidence [21], creation and development of interpersonal and group communication [22], managerial skills, and understanding the health environment [23]. From the participants' point of view, professional communication skills have been extracted as professional competencies. Also, critical evaluation skills, creative thinking, mastery of research methods and medical information resources, search skills, resource management, teamwork, patience, kindness, and motivation for success have been confirmed. Moreover, results of this study showed that mastery of new information technologies in the field of medicine will enhance the contribution of the librarian in the treatment team that would consequently lead to providing more effective services. To get fully mastered in these skills, the clinical librarian must have a deep knowledge of clinical terminology and English language; this will ultimately lead to the improvement of the process of supporting continuing medical education in the field of information resources and information retrieval skills.

Having advanced professional search skills in information resources, critical evaluation, and in-depth knowledge of clinical terms will enhance the confidence of the information specialist [18, 19] that cooperates as an expert in how to use databases and information related to health [24, 25]. In this regard, clinical librarian should be familiar with information organizations [22] and be capable of evaluating information and scientific texts [18, 25, 26]. To maintain this position and appear successfully in new roles, librarians need to have interaction and teamwork skills, gain the trust of the clinical team, be familiar with organizational policies in the clinical environment, and have interpersonal communication skills [12, 15]. The development of interdisciplinary and interpersonal relationships to work in a health-care environment has been considered a necessary and important need for clinical librarians [27].

Since the role of librarians in health and medical programs have been changed, the inclusion of health information literacy skills in the medical curricula [28], clinical culture, organizational policies, and relationships with team members, patients and their families in the clinical environment have been expressed as the educational needs of a clinical librarian [1]. To understand and play the extracted roles of a clinical librarian, they should acquire professional-interactive skills. According to the themes and subcategories of this study, it is recommended to develop an educational program based on the career and professional needs of clinical librarians and the prevailing culture of medical settings. To train a clinical librarian, educational needs such as computer skills, content production, information therapy, English language proficiency, familiarity with evidence-based medicine and information resources in this field, training courses in a clinical environment under the supervision of an instructor, and effective communication from the point of view of librarians are necessary to be able to interact with people in the clinical environment.

In order to meet these needs and develop training programs to acquire research skills, and skills in using technologies to organize and manage information, colleges and universities should offer training programs, including short-term courses related to clinical librarianship, to master's and doctoral students [29]. Colleges and specialized associations have to plan training courses to help clinical librarians develop skills and acquire the necessary competencies, including management skills, familiarity with new technologies in the field of health, understanding the health environment, and management and organization of health information which all are required to update professional knowledge [19, 23].

## Limitations

This study has some limitations. First, some participants have not been willing to participate in the interview. Although we attempted to assure this group of participants that their information would remain confidential, they still were unwilling to take part in interviews. Second, the interviewees selected to participate in the study were reluctant to collaborate due to their daily workload. With respect to this limitation, we attempted to schedule the interview sessions according to interviewees’ working schedule. Third, the sample recruitment of this study was limited to librarians who live in Tehran, capital of Iran; we think that there might be qualified librarians in other cities of the country that we were unable to recruit them to participate in this study.

## Conclusions

As the results showed, the clinical librarians have the role of providing information for the treatment team. They must be able to teach information literacy to medical staff, and health literacy to patients. Also, the clinical librarians should have a good mastery of professional skills. Curricula are an essential part of training clinical librarians for improving their job skills. Curricula should be modified in order to be fully in line with the librarians' professional needs in the medical environment. One of the strengths of this study was identifying the participants’ views toward developing regular training programs. In this regard, holding short-term training courses to empower librarians and setting up an independent academic department at medical universities across the country can be effective in accelerating the implementation of clinical librarians’ roles.

## Supplementary Information

**Additional file 1**. Semi-structured interview Guideline.

## Data Availability

The datasets used and/or analyzed during the current study are available from the corresponding author on reasonable request.

## Abbreviations

IUMS: Iran University of Medical Sciences
TUMS: Tehran University of Medical Sciences
LMI: Library and Medical Information
