# Correction to: Skills and key education needed for clinical librarians: an exploratory study from the librarians’ perspectives

**DOI:** 10.1186/s12911-021-01637-0

**Published:** 2021-10-04

**Authors:** Maryam Zarghani, Leila Nemati-Anaraki, Zahra Dinpajoo, Arezoo Ghamgosar, Sedegheh Khani, Maryam Khazaee-Pool

**Affiliations:** 1 School of Health Management and Medical Information Science, Iran University of Medical Sciences, Tehran, Iran; 2 Department of Medical Library and Information Sciences, School of Health Management and Medical Information Science, Iran University of Medical Sciences, Tehran, Iran; 3 Department of Public Health, School of Health, Mazandaran University of Medical Sciences, Sari, Iran; 4 Health Sciences Research Center, Addiction Research Institutes, Mazandaran University of Medical Sciences, Sari, Iran

## Correction to: BMC Med Inform Decis Mak (2021) 21:40 10.1186/s12911-021-01601-y

Following publication of the original article [1], it was reported that Arezoo Ghamgosar’s name was erroneously published as ‘Arezo Ghamgosar’.

The original article has been corrected.
