# Supplementary material for: Skills and key education needed for clinical librarians: an exploratory study from the librarians' perspectives
Source: BMC Med Inform Decis Mak. 2021 Aug 9;21:240. doi: 10.1186/s12911-021-01601-y (PMC8351442; doi:10.1186/s12911-021-01601-y)
Supplement: Supplementary file 1 — Additional file 1. Semi-structured interview Guideline. [file 12911_2021_1601_MOESM1_ESM.docx]

**Semi-structured interview Guideline (Supplementary file1)**

**Research details**

**Title:** Skills and Key Education for Clinical Librarians

**Ethics code**: IR.IUMS.REC.1397.669

**Clinical Librarian**: A Clinical Librarian is a professional and capable person who is effective in meeting the information needs of medical and research team members and has a key role in supporting clinical decisions made with high-quality information.

**Questions**

1. Tell me about yourself and give a brief summary of your professional and research background? (Educational level, job position, and research)

2. What is your opinion about the role of clinical librarian and where can it play a role?

3. What is the specialized and professional skills for the clinical librarian?

4. What are the communication skills needed for the clinical librarian?

5. What are the necessary training programs for the clinical librarians?

6. Is there anything that is not mentioned and you want to tell.
